# Adjuvant Therapy for Resectable Biliary Tract Cancer: A Bayesian Network Analysis

**DOI:** 10.3389/fonc.2021.600027

**Published:** 2021-03-11

**Authors:** Xiuqiong Chen, Fanqiao Meng, Hua Xiong, Yanmei Zou

**Affiliations:** ^1^Department of Oncology, Tongji Hospital, Tongji Medical College, Huazhong University of Science and Technology, Wuhan, China; ^2^Department of Hematology, Tianjin Medical University General Hospital, Tianjin, China

**Keywords:** biliary tract cancer (BTC), adjuvant therapy (AT), gemcitabine, fluorouracil, chemo-radiotherapy, radiotherapy, observation

## Abstract

**Background:** Selecting proper postoperative adjuvant therapy is of great importance for prolonging overall survival (OS) of patients with biliary tract cancer (BTC). OS is commonly affected by high rate of postoperative recurrence and metastasis.

**Purpose:** The present study aimed to identify the optimal adjuvant therapy for BTC patients.

**Method:** A comprehensive search was carried out on Pubmed, Web of science, and Embase databases to acquire articles regarding BTC therapy approaches. Subsequently, the hazard ratio (HR) and its 95% confidence intervals (CIs) were applied to evaluate the efficacy of different adjuvant therapy regimens. The GemTc (GemTc.0.8-2) and R (R.3.6.0) software were employed to perform statistical analyses.

**Result:** Data from 22 articles, including 14,646 patients, were quantitatively analyzed. The results showed that in terms of 5-year OS, gemcitabine (GEM) was considered as the optimal adjuvant therapy for BTC compared with chemoradiotherapy (CRT; HR = 0.59; 95% CI = 0.34-0.97), observation (OB; HR = 0.49; 95% CI = 0.33-0.73), and radiotherapy (RT; HR = 0.40; 95% CI = 0.22-0.71). Additionally, 5-fluorouracil (5-FU) exhibited improved efficacy compared with RT (HR = 0.52; 95% CI = 0.29-0.91) and OB (HR = 0.63; 95% CI = 0.43-0.92). When the efficacy of 5-FU was compared with that of GEM, the results showed that 5-FU (HR = 1.29) was more effective than GEM. Furthermore, CRT and RT prolonged positive resection margin (R^+^)-OS (HR = 0.69; 95% CI = 0.49-1.00) and positive lymph node-(N^+^)-OS (HR = 0.22; 95% CI = 0.074-0.66) in BTC patients. In terms of median recurrence-free survival (RFS) and 1-year OS, the differences were not statistically significant among different therapeutic interventions.

**Conclusion:** The present study suggested that GEM could be used as a first-line adjuvant therapy for resected BTC patients. Additionally, CRT could be the optimal treatment approach for R^+^ and N^+^ patients.

## Introduction

It is well-known that biliary tract cancer (BTC), including gallbladder carcinoma and cholangiocarcinoma, belongs to a collective category of cancers, which is an aggressive malignant tumor with increasing incidence worldwide, accounting for ~3% of gastrointestinal tumor cases (1, 2). Like many other gastrointestinal tumors, the majority of BTC patients are diagnosed at an advanced stage, therefore, only 20% of BTC patients are eligible to undergo radical resection (3, 4). However, even after radical resection, the 1-year recurrence rate has been estimated to be ~50% (4, 5). Therefore, there is an imperative need for effective postoperative adjuvant therapies, including radiotherapy (RT), chemotherapy, and chemoradiotherapy (CRT), in order to prolong the overall survival (OS) and disease-free survival (DFS) of BTC patients. Currently, several adjuvant therapy strategies have been developed, however, which type of adjuvant therapy offers the most optimal survival benefit remains still controversial.

Over the past decades, researchers around the world have attempted to develop an effective adjuvant therapy for BTC, however, no considerable progress has been achieved. Randomized controlled trials (RCTs) on BTC are still sparse, and this is especially true for studies on adjuvant therapy. However, some retrospective studies and review reports have confirmed the importance of adjuvant therapy. A meta-analysis, including 20 clinical trials, revealed that adjuvant therapy could not provide a survival benefit for patients with BTC, however, the benefits on positive lymph node (N^+^) and positive resection margin (R^+^) disease status were confirmed (6). Another study suggested that gemcitabine (GEM) was the appropriate adjuvant therapy for BTC, with a tolerable toxicity, while concurrent CRT offered short-term survival benefits following tumor resection (7). Recently, several prospective trials have been published regarding adjuvant therapy for BTC, thus providing powerful evidence for treatment options. Two randomized phase III studies, each including 225 and 196 patients, investigated whether GEM-based therapy could result in significantly increased OS and recurrence-free survival (RFS) rates compared with surveillance only. No obvious benefits were observed in GEM-treated patients despite the good tolerance (8, 9). Furthermore, BILCAP study, a randomized, controlled, multicenter, phase III clinical trial, demonstrated that capecitabine, as adjuvant chemotherapy, exhibited beneficial effect on OS and a manageable safety profile in patients with resected BTC (10). In the present study, a Bayesian network meta-analysis was performed based on all eligible publications, in order to identify the optimal adjuvant therapy for BTC.

## Materials and Methods

### Literature Search Strategy

In the present study, a systematic review of the English literature was performed on Pubmed, Web of Science, and EMBASE databases, until April 1, 2019, according to the Preferred Reporting Items for Systematic Reviews and Meta-Analyses (PRISMA) guidelines (11). The combinations of keywords used were as follows: “adjuvant treatment”; “adjuvant therapy”; “adjuvant chemoradiotherapy”; “adjuvant radiotherapy”; “adjuvant chemotherapy”; and “resected; “resectable”; and “cholangiocarcinoma”; “gallbladder cancer”; “biliary tract cancer”; “biliary cancer”; “bile duct cancer.” The reference lists of previous meta-analyses and published articles from the initial search were also screened in order to avoid omission of relevant literatures.

### Inclusion Criteria

The eligibility criteria were defined by the Population, Intervention, Comparison, Outcome, Study design (PICOS) framework (11). (i) Population: All postoperative studies, which defined patients as the target population. All patients were pathologically diagnosed with BTC; (ii) Intervention: Interventions included adjuvant CRT, adjuvant chemotherapy [5-fluorouracil (5-FU) and GEM], adjuvant RT and observation. Furthermore, at least 20 BTC patients were included in each intervention; (iii) Comparison: Each study was composed of at least two or more interventions and the comparison was performed between the interventions. (iv) Outcome: The main outcomes of interest included 1-year OS rate, 5-year OS rate, and median-RFS rate. Other data such as survival and recurrence were also available in the selected articles.

### Data Extraction and Quality Assessment

Data were extracted from the selected literature by two investigators independently (FM and YLW). Data included author, year, treatment measures, patient number, design scheme, 1-year OS rate, 5-year OS rate, median-RFS rate, and the efficacy of adjuvant therapy on N^+^ and R^+^ patients. The kappa coefficient (κ) was applied to evaluate the consistency of the data extracted by the two investigators (12). The SPSS software (SPSS 16.0) was used to calculate the κ value, while κ > 0.5 was considered to indicate a good consistency among data. If discrepancies emerged, a third investigator joined to resolve the disputes between the two investigators. If data from the extracted literatures were missing, the practical methods by Tierney et al. (13) and Parma et al. (14) were applied to analyze missing statistical variables. In addition, the Cochrane Collaboration's Risk of Bias (ROB) assessment tool was used to evaluate risk of bias and quality of RCTs (15), while the Newcastle-Ottawa Scale (NOS) was utilized to assess the quality and risk of bias of the non-RCTs (16, 17). Finally, the Grading of Recommendations Assessment, Development and Evaluation (GRADE) framework was used to assess the quality of evidence (18).

### Data Analysis

#### Heterogeneity

A pair-wise meta-analysis was performed to synthesize evidence from multiple studies with the same treatment regimen. When quantification of heterogeneity could not be performed, the fixed-effect or random-effect model was adopted. The Cochran's I square (I^2^) and Q statistics were used to determine the percentage of heterogeneity among studies (19, 20). The I^2^ value was mainly used to describe heterogeneity, and a value >50% was considered to indicate a statistically significant heterogeneity, suggesting that a subgroup analysis was required to identify the source of heterogeneity (21).

#### Consistency and Inconsistency

Unlike traditional meta-analysis, inconsistency in network meta-analysis refers to the similarity between direct and indirect results (22). Therefore, the node-splitting analysis in the R software was employed to calculate the inconsistency value between the direct and indirect results (23). A significant inconsistency was indicated when node-splitting analysis derived *P* < 0.05 in the Bayesian network meta-analysis. Finally, when the inconsistency was not statistically significant, a consistency model was then employed.

#### Network Meta-Analysis and Rank Probabilities

HR for 1-year OS, 5-year OS, and median-RFS rate with its corresponding 95% CI was used as the effect size estimate. To determine HRs and CIs, the number of deaths and sample size for each therapy from all studies were implemented into the GemTc software. When HR was <1.0 and a value = 1.0 was not contained in the 95% CI, the results were statistically significant, indicating that the intervention of the experimental group was more effective compared with that of the control group. Furthermore, the ranking graphs of different outcomes were obtained. In the present study, the Brooks-Gelman-Rubin method was utilized to calculate the Potential Scale Reduction Factor (PSRF). PSRF represents the convergence degree by comparing the within-chain and between-chain variance. Therefore, a PSRF value close or equal to 1.0 indicated that an optimal convergence was achieved. Additionally, the Markov chain Monte Carlo (MCMC) method based on the Bayesian framework was applied for simulation analysis. Herein, four different chains were set, each producing 50,000 simulation iterations with a thinning rate of 10. When 20,000 simulation iterations were completed, an optimal convergence degree was achieved (24, 25).

## Results

### Literature Features

**Figure 1:**
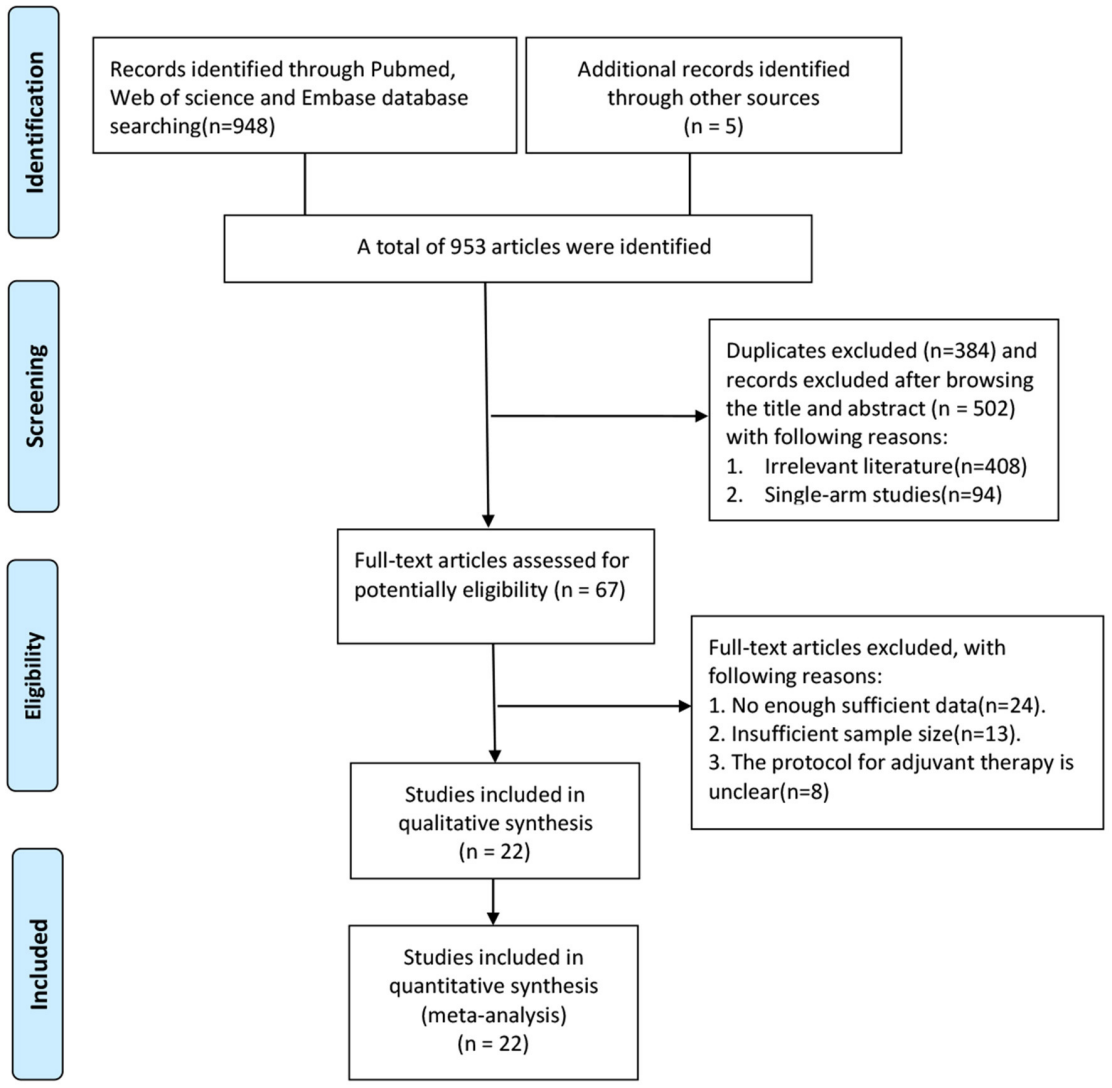
Flow chart of literature screening.

**Figure 2:**
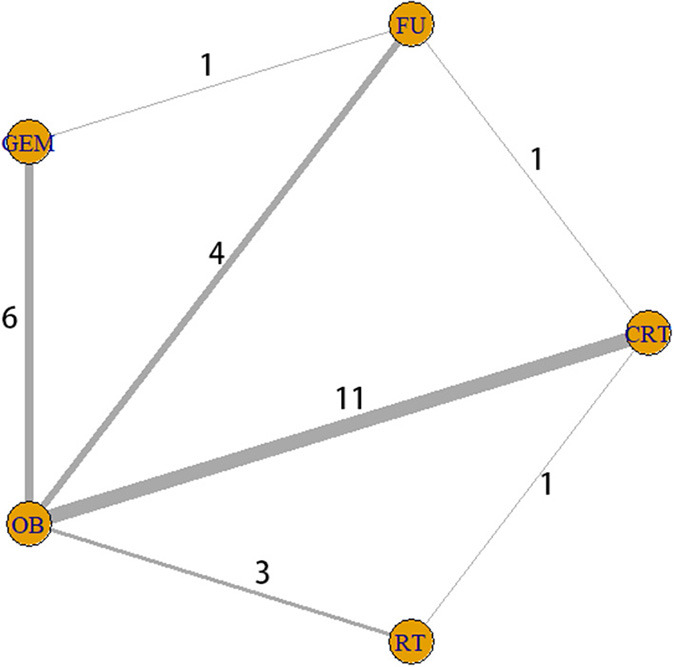
Interaction network between interventions. The five yellow circles represent five different interventions, while the thickness of the lines connecting the circles is proportional to the number of trials.

By searching different databases in detail, a total of 2,592 relevant articles were identified. Following check for duplicate articles, and preliminary screening of titles and abstracts, 2,525 references were excluded, and 67 potentially eligible literatures were assessed for full-text screening. Then, a total of 45 articles were excluded for the following reasons: insufficient data (24 articles); insufficient sample size (13 articles); and lack of detailed description of adjuvant therapy (8 articles). Finally, 22 articles with a total of 14,646 patients were selected for quantitative synthesis according to the filtering process shown in Figure 1. Patients with surgery alone accounted for 64.4% and adjuvant therapy accounted for 35.6%. Analysis using the SPSS software revealed a κ value equal to 0.507, indicating that the data extracted by the two investigators were consistent. In the current study, five interventions were categorized into seven different comparison groups, namely the GEM vs. OB, 5-FU vs. OB, RT vs. OB, CRT vs. FU, CRT vs. OB, CRT vs. RT, and GEM vs. 5-FU groups. All patients were treated with one of the five aforementioned therapies (Figure 2). Of the 22 studies we included, the dose of chemotherapy was not completely consistent according to the patient's condition in different studies. Thirteen studies involved radiotherapy, and 9 studies specified the radiotherapy dose (median radiation dose was 50.4Gy), only 2 studies specified radiotherapy type. In the 22 eligible studies, five RCTs (8–10, 26, 27) and 17 retrospective studies (28–44) were included. In addition, these studies were conducted in different countries, and more specifically, nine in the US (30, 33, 35–37, 40, 42, 43), six in Japan (9, 15, 27, 29, 33–35, 39–41, 45, 46), three in Korea (31, 32, 38), two in UK (10, 26), one in France (8) and one in India (44). The sample size in each study ranged from 25 to 5,739 patients. A total of 9,430 patients underwent BTC resection alone, while 5,216 patients were treated with surgery and adjuvant therapy. In terms of tumor sites, four studies involved two sites, including the bile duct and the gallbladder, 14 studies only the bile duct, and the remaining four the gallbladder. Finally, among the 22 studies, two were three-arm trials and the remaining 20 two-arm trials. All data are summarized in Table 1.

**Table 1:** Features of the included studies.

**References**	**Country**	**Tumor type**	**Therapy /Control**	**No. of patients**	**Study design**	**Clinical factors**
Edeline et al. (8)	France	Bile duct cancer and gallbladder cancer	GEM vs. OB	73/82	RCT	1-year OS median-RFS N(+)-OS R(+)-OS
Primrose et al. (10)	UK	Bile duct cancer and gallbladder cancer	FU vs. OB	223/ 224	RCT	5-year OS median-RFS N(+)-OS R(+)-OS
Ebata et al. (9)	Japan	Bile duct cancer	GEM vs. OB	117/ 108	RCT	5-year OS median-RFS
						N(+)-OS
						R(+)-OS
Leng et al. (28)	USA	Bile duct cancer	RT vs. OB	762/1155	Retro-	5-year OS
Mizuno et al. (29)	Japan	Bile duct cancer	GEM vs. OB	67/113	Retro-	5-year OS median-RFS
Go et al. (31)	Korea	Gallbladder cancer	CRT vs. FU	45/39	Retro-	5-year OS median-RFS N(+)-OS
Im et al. (32)	Korea	Bile duct cancer	CRT vs. OB vs. RT	49/168/29	Retro-	5-year OS median-RFS R(+)-OS
Dover et al. (30)	USA	Bile duct cancer	CRT vs. OB	23/72	Retro-	R(+)-OS
Wang et al. (33)	USA	Gallbladder cacner	CRT vs. OB	68/44	Retro-	5-year OS median-RFS
Toyoki et al. (34)	Japan	Bile duct cancer	FU vs. OB	55/99	Retro-	5-year OS
Hoehn et al. (35)	USA	Bile duct cancer	CRT vs. OB	1902/5739	Retro-	N(+)-OS R(+)-OS
Hyder et al. (36)	USA	Gallbladder cancer	RT vs. OB	894/894	Retro-	1-year OS 5-year OS
Neoptolemos et al. (26)	UK	Bile duct cancer	GEM vs. OB vs. FU	146/145/143	RCT	1-year OS 5-year OS
Narang et al. (37)	USA	Bile duct cancer	CRT vs. OB	66/120	Retro-	5-year OS median-RFS
Kim et al. (38)	Korea	Bile duct cancer	CRT vs. OB	115/53	Retro-	5-year OS median-RFS
Murakami et al. (39)	Japan	Bile duct cancer	GEM vs. OB	49/78	Retro-	1-year OS 5-year OS
Gold et al. (40)	USA	Gallbladder cancer	CRT vs. OB	25/48	Retro-	1-year OS 5-year OS median-RFS
Murakami et al. (41)	Japan	Bile duct cancer and gallbladder cancer	GEM vs. OB	50/53	Retro-	1-year OS 5-year OS
Borghero et al. (42)	USA	Bile duct cancer	CRT vs. OB	42/23	Retro-	5-year OS median-RFS
Hughe et al. (43)	USA	Bile duct cancer	CRT vs. OB	34/30	Retro-	1-year OS 5-year OS
Sikora et al. (44)	India	Bile duct cancer	CRT vs. OB	49/55	Retro-	1-year OS 5-year OS
Takada et al. (27)	Japan	Gallbladder cancer	5-FU vs. OB	69/43	RCT	1-year OS 5-year OS median-RFS
		Bile duct cancer	5-FU vs. OB	82/84	RCT	1-year OS 5-year OS median-RFS

### Quality Assessment

**Figure 3:**
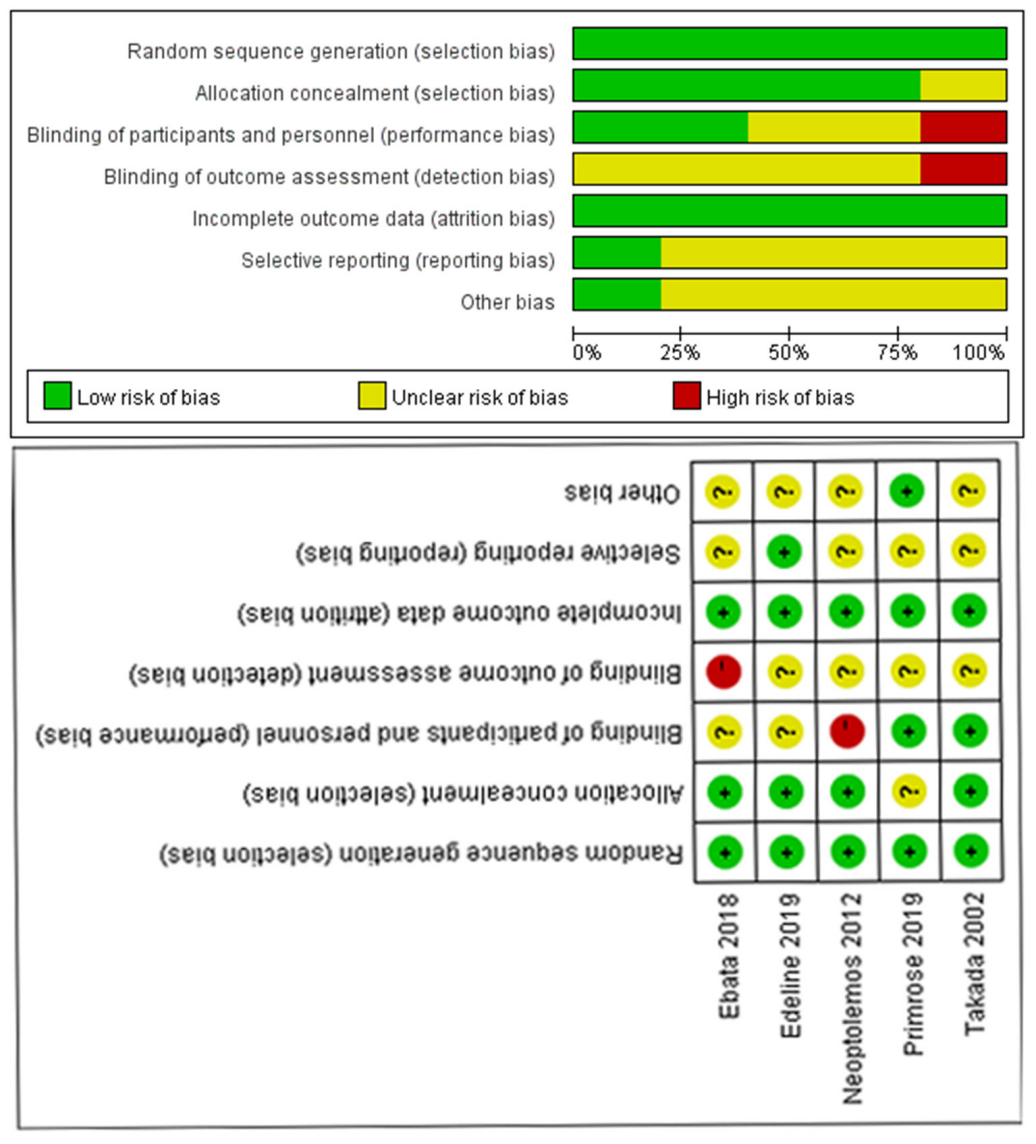
Quality assessment chart.

Following the selection of eligible studies, the quality of the included studies was subsequently evaluated. Therefore, the Cochrane Collaboration's tool, covering five domains of bias, namely the selection bias, performance bias, attrition bias, measurement bias, and reporting bias, was applied to evaluate RCT risk of bias and quality of evidence. In Figure 3, green, yellow, and red represents low, unclear, and high risk of bias, respectively. The analysis revealed that all five RCTs exhibited high quality and low risk of bias (Figure 3). Furthermore, NOS was employed to assess the quality of retrospective studies. In NOS, three domains of evaluation of the risk of bias are included, namely the research subject selection, intergroup comparability, and measurement of exposure factors. A maximum of nine points are available, and a total score ≥6 is considered to indicate high quality. Herein, NOS results demonstrated that all studies displayed a score of ≥6 (Table 1), thus suggesting that the quality of all retrospective studies was high. The quality rate of RCTs is summarized in Table 2.

**Table 2:** Quality assessment of retrospective studies.

**References**	**Is the case definition adequate?**	**Representativeness of cases**	**Selection of Controls**	**Definition of Controls**	**Comparability of cases and controls on the basis of the design or analysis**	**Ascertainment of exposure**	**Same method of Ascertainment for cases and controls**	**Non-Response**	**Total score**
Leng et al. (28)	1	1	1	0	1	1	1	1	7
Mizuno et al. (29)	1	1	1	0	2	1	1	1	8
Go et al. (31)	1	1	1	1	1	1	1	0	7
Im et al. (32)	1	1	1	1	1	1	1	1	8
Dover et al. (30)	1	1	1	0	1	1	1	1	7
Wang et al. (33)	1	1	1	0	1	1	1	1	7
Toyoki et al. (34)	1	1	1	0	1	1	1	1	7
Hoehn et al. (35)	1	1	1	0	1	1	1	1	7
Hyder et al. (36)	1	1	1	1	1	1	1	0	7
Narang et al. (37)	1	0	1	1	1	1	1	0	6
Kim et al. (38)	1	1	1	0	1	1	1	0	6
Murakami et al. (39)	1	1	1	0	1	1	1	1	7
Gold et al. (40)	1	1	1	0	1	1	1	0	6
Murakami et al. (41)	1	1	1	0	1	1	1	0	6
Borghero et al. (42)	1	1	1	1	1	1	1	1	8
Hughe et al. (43)	1	1	1	0	1	1	1	1	7
Sikora et al. (44)	1	1	1	1	1	1	1	1	8

### Evaluation of Heterogeneity

Subsequently, the heterogeneity among the selected studies was evaluated based on three outcome indicators, namely the 1-year OS rate, 5-year OS rate, and median-RFS rate (Table 2). For 1-year OS rate, the I^2^ value for each comparison group, namely the 5-FU vs. OB (I^2^ = 0%), RT vs. OB (I^2^ = 0%), CRT vs. OB (I^2^ = 0%), and GEM vs. OB (I^2^ = 36.1%) group, was <50%. Additionally, for 5-year OS rate, the I^2^ values for the 5-FU vs. OB, and CRT vs. OB comparison groups were 35.2 and 0%, respectively. For median-RFS rate, the I^2^ value of the 5-FU vs. OB, and RT vs. OB comparison groups was 0% for both groups. The aforementioned results indicated a low heterogeneity among groups. By contrast, in the RT vs. OB comparison group, the I^2^ value was 69.1% for the 5-year OS rate, supporting a high heterogeneity (Table 3). Subsequently, a subgroup analysis, based on tumor site, revealed that heterogeneity was reduced to 0%, suggesting that tumor site was one of the major factors affecting heterogeneity. When the studies were divided into RCTs and non-RCTs, the I^2^ value of the GEM vs. OB comparison group was decreased from 76.7 to 50% in terms of 5-year OS rate, and from 84.7 to 0% in terms of median-RFS rate. These findings confirmed the effect of research design on heterogeneity. In addition, when study location was used as a variable for subgroup analysis, the I^2^ value of the CRT vs. OB comparison group was decreased from 53.8 to 41.2% in terms of median-RFS rate, suggesting that geographical distribution could also contribute to heterogeneity among studies.

**Table 3:** Heterogeneity assessment among studies in terms of different outcomes.

**Category**	**1-year OS(*I^**2**^*)**	**5-year OS*(I^**2**^)***	**Median-RFS*(I^**2**^*)**
GEM vs. OB	36.10%	76.70%	84.70%
CRT vs. OB	0.00%	0.00%	53.80%
RT vs. OB	0.00%	69.10%	0.00%
FU vs. OB	0.00%	35.20%	0.00%

### Consistency Assessment and Network Analysis

**Figure 4:**
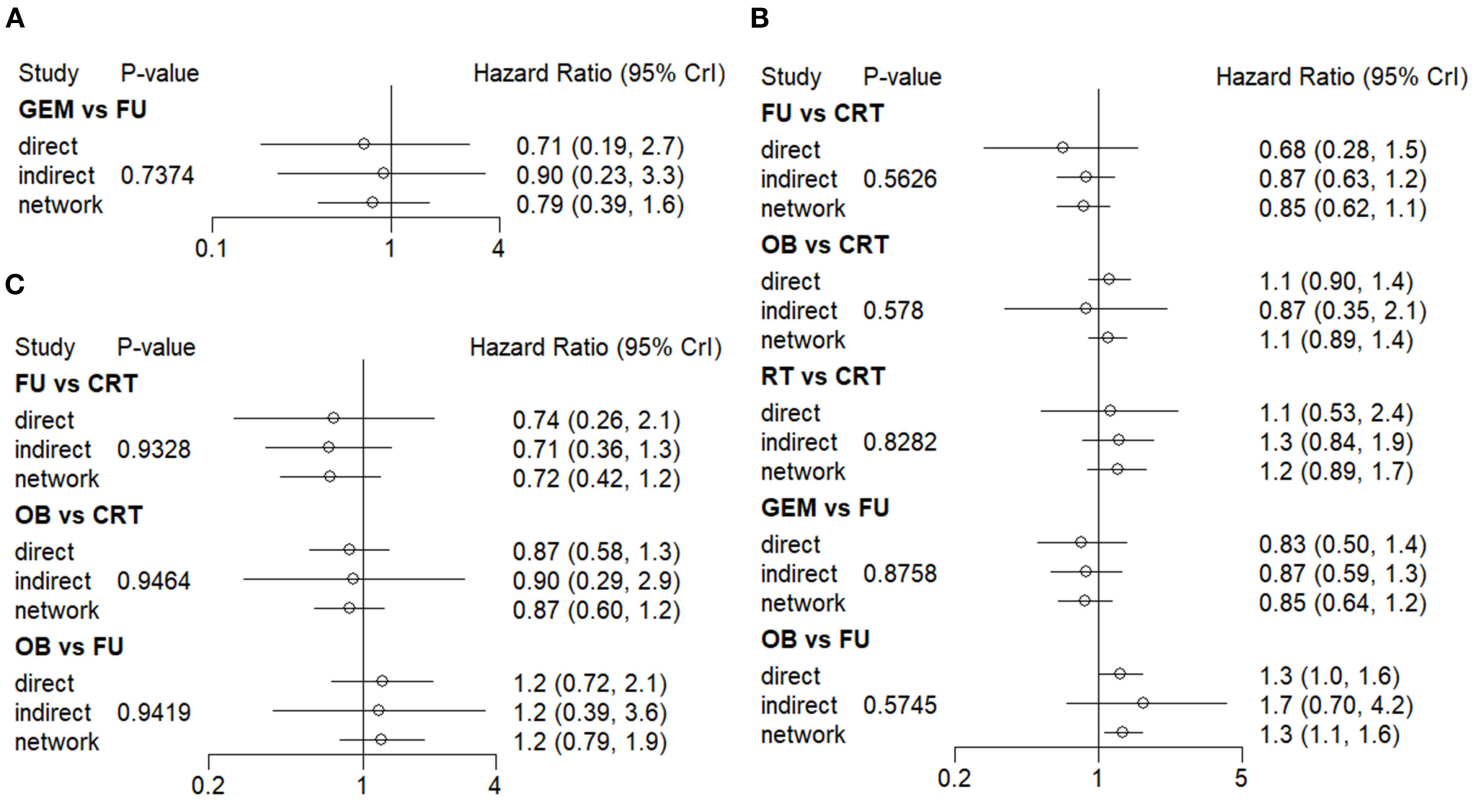
Consistency evaluation using forest plots. The **(A)** 1-year OS, **(B)** 5-year OS, and **(C)** median-RFS rates are presented. OS, overall survival; RFS, recurrence-free survival.

Both direct and indirect comparisons in the GEM vs. 5-FU group were then carried out, and the results showed that Bayesian *P* value was >0.05 in terms of 1-year OS rate (Figure 4A). Additionally, in different comparison groups, the *P* values of direct and indirect comparisons were also >0.05 in terms of the 5-year OS rate and median-RFS rate (Figures 4B,C). Therefore, no significant inconsistencies were observed between direct and indirect comparisons.

**Figure 5:**
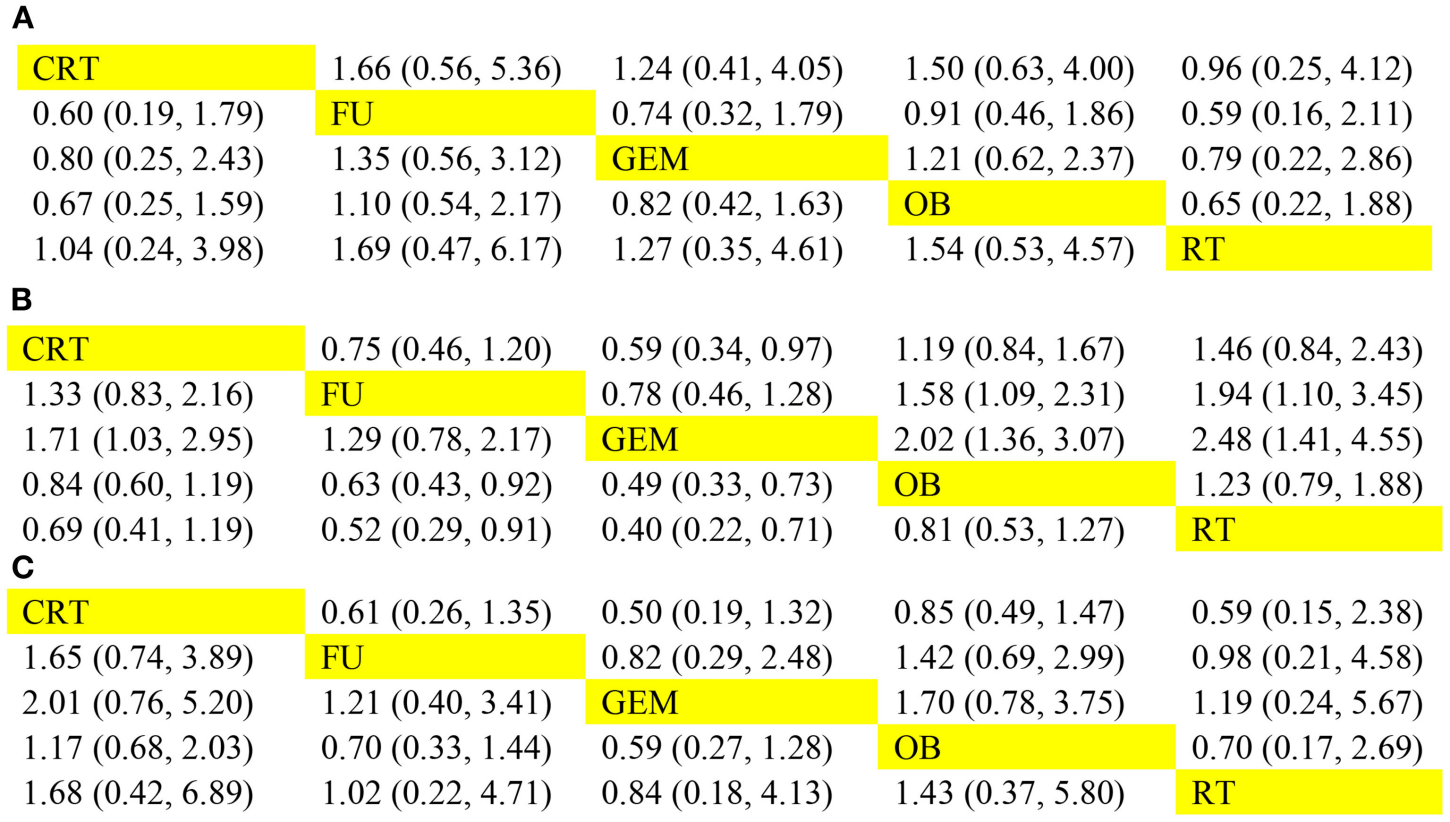
Comparison of efficacy among five adjuvant therapies. The pooled hazard ratios for the **(A)** 1-year OS, **(B)** 5-year OS, and **(C)** median-RFS rates are presented. The data in the upper right part represent the results obtained when the treatment on the row was compared with that in the column, while the data in the lower-left part represent the results obtained when the treatment on the column was compared with that on the row. OS, overall survival; RFS, recurrence-free survival.

**Figure 6:**
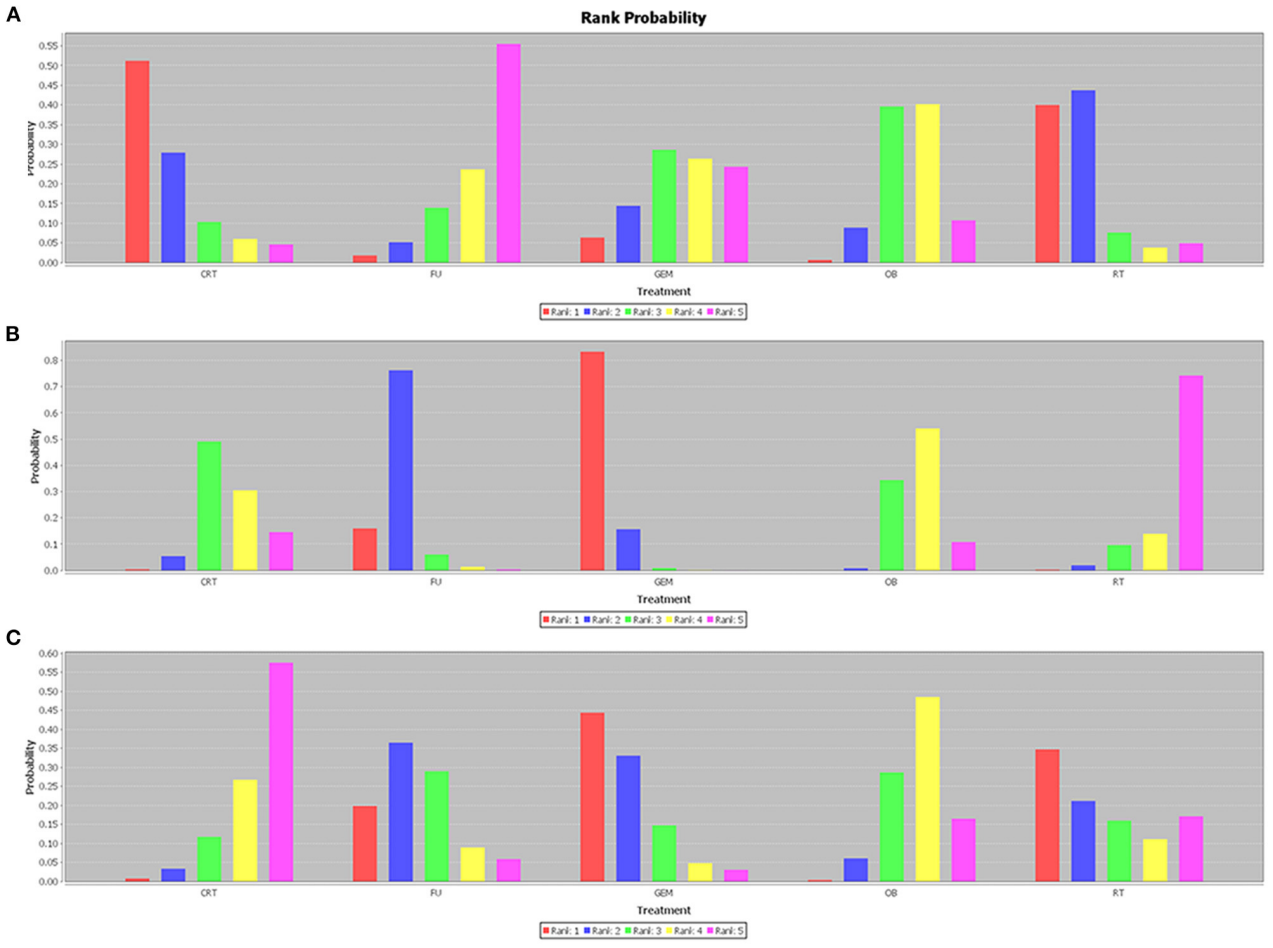
Efficacy rank histogram of different outcomes. The **(A)** 1-year OS, **(B)** 5-year OS, and **(C)** median-RFS rates are presented. Different colors correspond to different efficacy ranking. The higher the red column, the more likely to rank first. OS, overall survival; RFS, recurrence-free survival.

The network analysis results are presented in Figure 5. For 1-year OS rate, no statistically significant differences were observed between adjuvant therapy and OB (Figure 5A). More importantly, the analysis revealed that treatment with RT as adjuvant therapy could prolong the 1-year OS rate, compared with GEM (HR = 0.79), 5-FU (HR = 0.59), OB (HR = 0.65), and CRT (HR = 0.96). However, when the efficacy of different adjuvant therapies was ranked without considering toxicity, the effectiveness of CRT (51%) was higher compared with RT (40%), in terms of 1-year OS rate (Figure 6A). Additionally, GEM exhibited a significantly higher 5-year OS rate compared with CRT (HR = 0.59; 95% CI = 0.34-0.97), OB (HR = 0.49; 95% CI = 0.33-0.73), and RT (HR = 0.40; 95% CI = 0.22-0.71; Figure 5B). In terms of 5-year OS rate, 5-FU displayed better efficacy compared with OB (HR = 0.63; 95% CI = 0.43-0.92) and RT (HR = 0.52; 95% CI = 0.29-0.91), and worse compared with GEM (HR = 1.29; 95% CI = 0.78-2.17). No statistically significant differences were observed among the remaining comparison groups. As shown in Figure 6B, GEM was more likely to rank first for 5-year OS rate (83%). Furthermore, a detailed analysis was performed to determine whether adjuvant therapy could provide benefits in median-RFS rate. Therefore, compared with other adjuvant therapies, GEM displayed a greater tendency to provide median-RFS benefits (Figures 5C, 6C).

**Figure 7:**
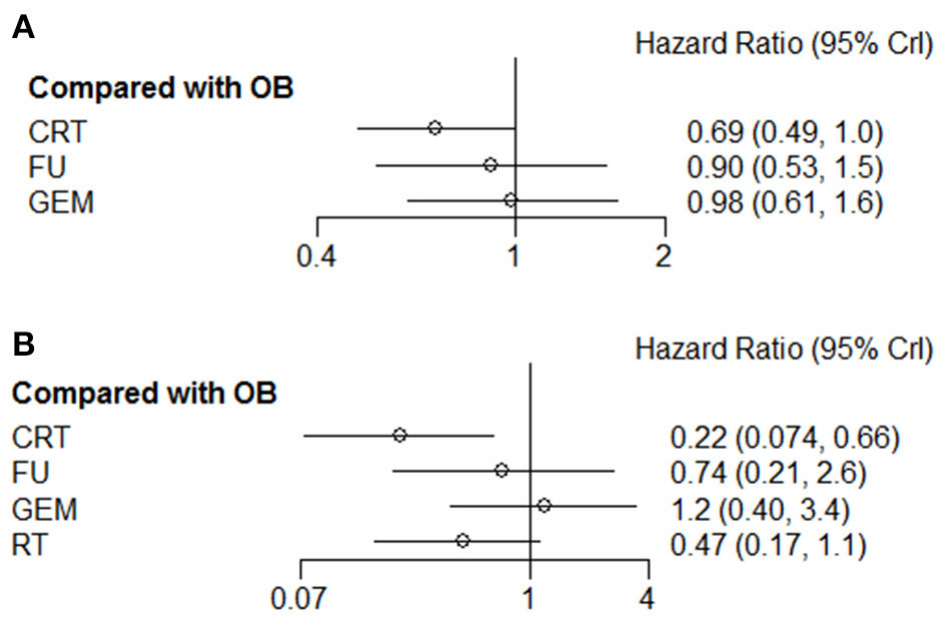
Pooled hazard ratios for OS of patients with **(A)** N^+^ or **(B)** R^+^ by Bayesian network analysis. OS, overall survival; N^+^, positive lymph node; R^+^, positive resection margin.

Given that tumors of the gallbladder and bile duct are etiologically different, it is more likely to respond differently to treatment. Therefore, the tumor sites were divided into different subgroups for analysis. The results revealed that for patients with cholangiocarcinoma, GEM ranked first (63%) among these interventions, and was the only effective therapy that could prolong the 5-year OS rate compared with OB (HR = 0.55; 95% CI = 0.34-0.91). There were no statistically significant differences among the remaining comparison groups. Regarding gallbladder cancer, CRT (74%) and 5-FU (85%) therapies were more likely to prolong 1-year and 5-year OS, respectively. However, the comparison between these results did not reach statistical significance. R^+^ and N^+^ are considered the most important risk factors for tumor recurrence, therefore, adjuvant therapy is particularly pivotal. The results of the comprehensive analysis showed that postoperative CRT could significantly improve the OS rate of N^+^ (Figure 7A) and R^+^ (Figure 7B) patients (HR = 0.69; 95% CI = 0.49-1.00; and HR = 0.22; 95% CI = 0.074-0.66).

### Publication Bias

**Figure 8:**
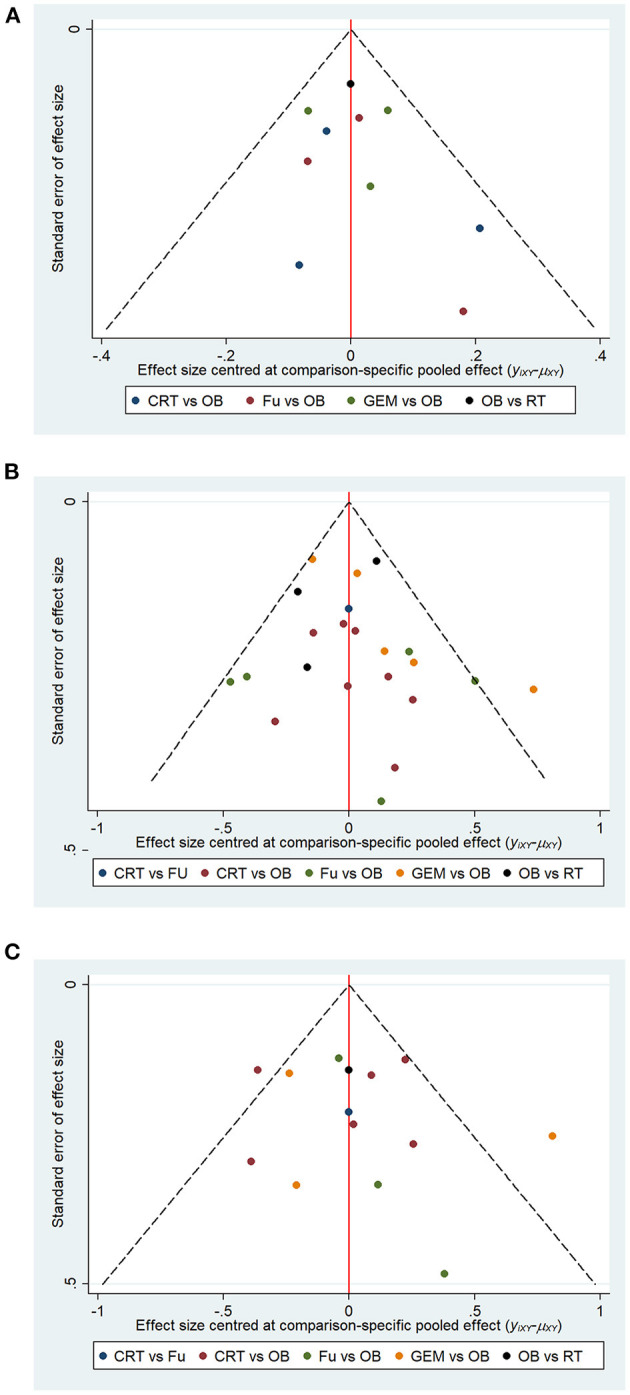
Funnel plots represent publication bias in terms of **(A)** 1-year OS, **(B)** 5-year OS, and **(C)** median-RFS rate. Different color dots represent different comparisons. The red line in the middle represents the null hypothesis, where the specific effect values in the trial are similar to that of the pooled effect sizes in comparisons.

The meta-funnel method was used to evaluate publication bias. The asymmetry of funnel plots for 1-year OS rate (Figure 8A), 5-year OS rate (Figure 8B), and median- RFS (Figure 8C) reflected the evidence of publication bias. The different colors in the funnel plots represent different comparison pairs. The dots beyond the slashes on both sides represent studies with a small sample size, which may exaggerate the effect of interventions. The results did not reveal any evidence of publication bias in the current study.

### Quality of the Evidence

In the present study, the quality of the selected five RCTs was moderate, and all risks of bias were considered low to moderate. The quality score of the 17 retrospective studies was ≥6, indicating good quality. However, the small number of head-to-head comparisons contributed to a serious inaccuracy, which affected the strength of inferences within the Bayesian network. In terms of heterogeneity, the GEM vs. OB comparison group presented higher heterogeneity for different outcomes, which also affected the evidence strength. Therefore, a subgroup analysis was carried out to reveal the sources of heterogeneity. Little evidence of inconsistency was observed between the direct and indirect assessments for the majority of comparisons (*P* > 0.05). It is well known that small sample-sized studies may affect the publication bias. However, in the present study only three small sample-sized trials, beyond the slashes on both sides of the funnel plots, contributed to publication bias. Overall, the strength of evidence was considered moderate to high, supporting the efficacy of GEM and 5-FU in extending 5-year OS compared with OB.

## Discussion

Currently, the research on adjuvant therapy for BTC is a hot issue, therefore, the association between adjuvant therapy and clinical outcome has been widely investigated. Since the existing trials are often small-sized and their findings are controversial, it remains difficult to determine whether resected BTC patients should undergo adjuvant therapy, and which type of therapy is more effective. Herein, a total of 22 studies, including 5 RCTs and 17 non-RCTs, were included in the Bayesian network analysis. The first-ranking adjuvant therapy was selected among seven comparison groups, namely the GEM vs. OB, 5-FU vs. OB, RT vs. OB, CRT vs. 5-FU, CRT vs. OB, CRT vs. RT, and GEM vs. 5-FU groups. The results of the current study provided strong evidence that both GEM and 5-FU, as adjuvant therapies, could provide BTC patients with long-term survival benefits, while GEM tended to exhibit a better efficacy compared with 5-FU (HR = 0.78; 95% CI = 0.46-1.28). In addition, for patients with N^+^ and R^+^, CRT could increase the OS rate compared with OB (HR = 0.69; 95% CI = 0.49-1; and HR = 0.22; 95% CI = 0.074-0.66), whereas RT could not provide any survival advantage.

CRT regimen for R^+^ patients has been widely adapted in clinical practice. However, for N^+^ patients, the available findings regarding the choice of chemotherapy or chemoradiotherapy remain controversial. Numerous studies have reported that R^+^ and N^+^ are negative predictors of survival for BTC patients (45–48). Based on these findings, Horgan et al. (6) conducted a meta-analysis to determine the effect of adjuvant therapy on survival rate. Consistent with the findings of the current study, the subgroup analysis in the above study showed that treatment with CRT (OR = 0.39; 95% CI = 0.61-0.98) adjuvant therapy could provide survival advantage in N^+^ (OR = 0.49; *P* = 004) and R^+^ (OR = 0.36; *P* = 002) patients, thus supporting the effective role of CRT as adjuvant therapy. However, the meta-analysis by Horgan et al. (6) classified all different treatments into the same class and no indirect comparison between treatments. Another analysis concluded that adjuvant chemotherapy administration gave an OS benefit in resected BTC, but this study didn't include treatment measures other than chemotherapy (49). Herein, all primary therapies (GEM, 5-FU, CRT, RT, and OB) were simultaneously compared, and the effect of each therapy was evaluated individually. Inconsistent with our results, another study showed that treatment with CRT did not provide long-term survival benefits for N^+^ (HR = 2.10; 95% CI = 0.31-14.34) and R^+^ patients (HR = 0.58; 95% CI = 0.06-6.17)(7). Such inconsistency could be attributed to more credible and robust data included in the current study. Therefore, our results could offer clinicians the necessary knowledge for selecting the appropriate adjuvant therapy for N^+^ and R^+^ patients.

The Bayesian analysis demonstrated that GEM and 5-FU could decrease mortality in patients with resected-BTC. This finding was partially consistent with a previous study conducted by Zhu GQ et al., indicating that intravenous GEM was closely associated with prolonged survival. The above network meta-analysis aimed to investigate the association between adjuvant therapy and survival, demonstrating that that GEM after surgery had manageable toxicity, and could significantly prolong survival of BTC patients (HR = 2.12; 95% CI = 1.23-4.02; *P* = 0.01) (7). Furthermore, the BILCAP trial was the only RCT to reveal a significant difference on OS in the 5-FU vs. OB comparison group (HR = 0.75; 95% CI = 0.58-0.97; *P* = 0.028), using a per-protocol analysis. However, in terms of unadjusted intention-to-treat (ITT) OS, no statistically significant differences were observed (HR = 0.81; 95% CI = 0.63-1.04; *P* = 0.097). Due to the inconsistency between ITT and per-protocol analysis, the quality of evidence was considered moderate to strong. In addition, the RCT by Ebata et al. (9) and the PRODIGE12 study reported similar results. Therefore, there was no significant difference in survival probability between the GEM and OB group in patients with resected-BTC (8, 9). This finding could be attributed to small sample size and decreased event rate. The present study was the first to assess the survival benefits, by performing direct and indirect comparisons among five different adjuvant therapies. Additionally, the results supported the conclusion that both GEM and 5-FU could provide survival advantages compared with OB, while GEM was more effective than 5-FU (HR = 0.78). However, head-to-head studies are still lacking. In Europe, the first head-to-head trial has currently begun, employing GEM in the experimental group, and patients switching from OB to 5-FU in the control group. The aforementioned trial could provide substantial evidence supporting the treatment of resected BTC patients with GEM or 5-FU (50). Herein, the Bayesian analysis with the strongest clinical evidence also provided a reference for the selection of appropriate treatment strategies.

The current study summarized all the reliable large sample-sized retrospective studies and RCTs in recent years, regarding the effect of different types of adjuvant therapy on BTC patients, by acquiring more comprehensive data compared with previous studies. The results could provide clinicians with the necessary knowledge for selecting the appropriate adjuvant therapy for BTC patients. The Bayesian network analysis also helped to avoid unnecessary selection bias by incorporating all present data into a single analysis (51). When no head-to-head trials are available, network meta-analysis is of great importance, since all indirect and direct comparison results can be combined in order to achieve a more accurate evaluation of the result (52). However, there are still some limitations in this study. For example, only five RCTs were included in the analysis, while the rest were retrospective studies. Secondly, no gray literature sources were searched. In addition, toxicity analysis was not carried out since most selected studies lacked data on adverse reactions. When data on adverse reactions were reported, these could not be included in the analysis due to high heterogeneity. Fourthly, data on lymph nodes and margins are still scarce in the published literature. In addition, data on surgical methods, different radiation and chemotherapy doses are insufficient, hierarchical analysis cannot be conducted. Finally, assessments on lymph node and margin status varied due to operation quality.

## Conclusion

In summary, this network analysis indicated that compared with surgery alone, adjuvant therapy included GEM- and 5-FU-based chemotherapy schemes could prolonged OS in BTC patients. Furthermore, GEM was more effective than 5-FU, and provided benefits on RFS rate. For N^+^ and R^+^ patients, CRT performance was associated with prolonged OS. In addition, the difference between adjuvant therapy and OB was not statistically significant in terms of short-term survival time, however, treatment with CRT or RT could improve survival of BTC patients.

## Data Availability Statement

The original contributions presented in the study are included in the article/supplementary files, further inquiries can be directed to the corresponding author/s.

## Author Contributions

XC and FM designed the research study, performed the analyses, and wrote the manuscript. YZ and HX critically revised the manuscript, performed and ensured correct analysis of the data. All authors contributed to the article and approved the submitted version.

## Conflict of Interest

The authors declare that the research was conducted in the absence of any commercial or financial relationships that could be construed as a potential conflict of interest.
